# Biomechanical Analysis of Double‐Level Oblique Lumbar Fusion with Different Types of Fixation: A Finite Element‐Based Study

**DOI:** 10.1111/os.13703

**Published:** 2023-04-18

**Authors:** Kaibin Fan, Di Zhang, Rui Xue, Wei Chen, Zhiyong Hou, Yingze Zhang, Xianzhong Meng

**Affiliations:** ^1^ Department of Spinal Surgery The Third Hospital of Hebei Medical University Shijiazhuang China

**Keywords:** Biomechanical, Cortical Bone Trajectory Screw, Finite Element Analysis, Oblique Lumbar Interbody Fusion, Osteoporosis, Posterior Pedicle Screw

## Abstract

**Objective:**

One well‐liked less invasive procedure is oblique lumbar interbody fusion (OLIF). The biomechanical characteristics of double‐level oblique lumbar interbody fusion in conjunction with various internal fixations are poorly understood. The purpose of this study was to clarify the biomechanical characteristics of double‐level oblique lumbar interbody fusion for osteoporosis spines using various internal fixation techniques.

**Methods:**

Based on CT scans of healthy male volunteers, a complete finite element model of osteoporosis in L1–S1 was established. After validation, L3–L5 was selected as the surgical segment to construct four surgical models: (a) two stand‐alone cages (SA); (b) two cages with unilateral pedicle screws (UPS); (c) two cages with bilateral pedicle screws (BPS); and (d) two cages with bilateral cortical bone trajectory screws (CBT). Segmental range of motion (ROM), cage stress, and internal fixation stress were studied in all surgical models and compared with the intact osteoporosis model.

**Results:**

The SA model had a minimal reduction in all motions. The CBT model had the most noticeable reduction in flexion and extension activities, while the reduction in the BPS model was slightly less than that in the CBT model but larger than that in the UPS model. The BPS model had the greatest limitation in left–right bending and rotation, which was greater than the UPS and CBT models. CBT had the smallest limitation in left–right rotation. The cage stress of the SA model was the highest. The cage stress in the BPS model was the lowest. Compared with the UPS model, the cage stress in the CBT model was larger in terms of flexion and LB and LR but slightly smaller in terms of RB and RR. In the extension, the cage stress in the CBT model is significantly smaller than in the UPS model. The CBT internal fixation was subjected to the highest stress of all motions. The BPS group had the lowest internal fixation stress in all motions.

**Conclusions:**

Supplemental internal fixation can improve segmental stability and lessen cage stress in double‐level OLIF surgery. In limiting segmental mobility and lowering the stress of cage and internal fixation, BPS outperformed UPS and CBT.

## Graphical abstract

A complete osteoporosis model was established.

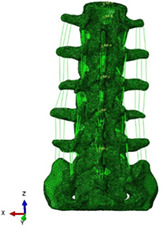

## Introduction

Lumbar interbody fusion (LIF) has been widely used for the treatment of lumbar degenerative disc disease in clinics. In recent years, minimally invasive spine surgery techniques have rapidly developed, such as oblique lateral interbody fusion (OLIF). Its advantages include less blood loss, shorter recovery time, less postoperative pain, and low incidence of associated neurological complications.^1^, ^2^, ^3^ Compared with the conventional posterior fusion cage, OLIF allows for the use of a larger intervertebral fusion cage, and because the fusion cage can be implanted across the epiphyseal ring of the vertebral body, its biomechanical stability is significantly improved.^4^, ^5^ Thus, stand‐alone OLIF has been applied in clinical practice and achieved good efficacy.^6^, ^7^ However, lumbar degenerative diseases occur mostly in elderly patients with varying degrees of bone loss, which increase the risk of cage subsidence.^8^, ^9^ To prevent subsidence and pseudoarthrosis, the OLIF technique is often used in clinical practice in combination with different internal fixation devices to increase the rigidity of surgical segments. There are many options for internal fixation; understanding which type of internal fixation offers superior biomechanical characteristics is currently a hot research topic. Many scholars have performed biomechanical finite element analysis of OLIF in combination with different internal fixation methods. Studies have shown that stand‐alone OLIF does not provide sufficient stability.^10^, ^11^ With the increase in the degree of osteoporosis, stand‐alone OLIF increased the potential risk of implant subsidence.^12^ The finite element biomechanical study showed that BPS provides the best biomechanical properties for OLIF,^10^, ^13^ and the lateral locking plate system and UPS can be used as alternatives to BPS in OLIF.^13^, ^14^ However, most OLIF biomechanical studies are limited to a single segment. There is a lack of literature evaluating the need for supplementary instrumentation after double‐level OLIF.

Finite element (FE) analysis has been used in biomechanical research for decades because of its reliability, convenience, and repeatability.^15^, ^16^ In this study, we used FE analysis to evaluate the biomechanical stability of double‐level stand‐alone OLIF versus double‐level OLIF with several types of supplemental instrumentation. The purpose of this study was: (i) to compare the biomechanical properties of different fixation methods; (ii) to analyze the factors associated with cage subsidence; and (iii) to determine which fixation method has the best biomechanical performance in the osteoporosis model.

## Methods

### Construction of an Intact Lumbar Finite Element Model

In this study, a 30‐year‐old healthy male volunteer with a height of 178 cm and weight of 81 kg was selected for a CT scan with a slice thickness of 0.625 mm, excluding a history of spinal deformity and lumbar disease. A total of 570 CT scan images were stored in DICOM format. The CT scan images were processed using commercial software (Mimics 21.0; Materialize, Leuven, Belgium) to create a 3D model. The model was reconstructed using reverse engineering software (Geomagic studio 12.0; Geomagic Inc., North Carolina, USA). Subsequently, models of soft tissue structures such as intervertebral discs were established by Croe8.0 software. After repair, the FE meshes of the different lumbar components were constructed using computer‐aided engineering (ANSA) software (BETA CAE Systems S. A, Thessaloniki, Greece). Finally, biomechanical simulation and results analysis were carried out by finite element analysis software (Abaqus, Simulia, Providence, RI, USA). The finite element model (FEM) construct comprised L1–S1 vertebrae, posterior elements, end‐plates, intervertebral discs, and the ligament system. The thickness of the cortical bone was 1 mm.^17^ The intervertebral discs consist of the annulus fibrosus, nucleus pulposus, and superior and inferior end‐plates. The discs were defined to be composed of 44% nucleus pulposus and 56% annulus fibrosus based on histological data,^18^ and the thickness of the end‐plate was 0.5 mm.^19^ The ligaments consisted of the anterior longitudinal ligament, the posterior longitudinal ligament, the ligamentum flavum, the interspinous ligament, the supraspinal ligament, the capsular ligaments, and the intertransverse ligament. They were set as truss elements (T3D2) bearing tensile loads only. The FEM was meshed using tetrahedral and hexahedral elements, except for the ligaments. The material properties of the components are shown in Table 1.^16^, ^20^, ^21^, ^22^ The osteoporosis model was established by simulating the loss of elastic modulus of normal bone, with the elastic modulus of cortical and cancellous bones decreasing by 33% and 66%, respectively.^23^

**TABLE 1:** Material properties of the FEM and implants

Components	Young's modulus (MPa)	Poisson ratio
Cortical bone	12,000	0.3
Cancellous bone	100	0.2
End‐plate	4000	0.3
Nucleus pulposus	1	0.49
Annulus	4.2	0.45
Anterior longitudinal ligament	20	0.3
Posterior longitudinal ligament	20	0.3
Ligamentum flavum	19.5	0.3
Interspinous ligament	11.6	0.3
Supraspinous ligament	15	0.3
Transverse ligament	58.7	0.3
Capsular ligament	32.9	0.3
Cage	3500	0.3
Screw and rod	110,000	0.3

### Construction of the Surgical Finite Element Model

Three‐dimensional geometric models of internal fixation instrumentation were constructed based on the actual parameters of the interbody cage and supplemental fixations using the part interface of Creo8.0. The interbody cage was modeled based on the Oracle cage (DePuy Synthes). It was 40 mm long, 22 mm wide, 11 mm high in front, 8 mm high in back, and had an 8° lordosis. The diameter of the pedicle screw was 6.5 mm, and the length was 50 mm. The diameter of the cortical bone screw was 5.0 mm, and the length was 30 mm. The diameter of the rod was 5.5 mm. The cage and supplemental fixations were selected for tetrahedral mesh processing. The detailed material properties of each component are listed in Table 1.

**Fig. 1:**
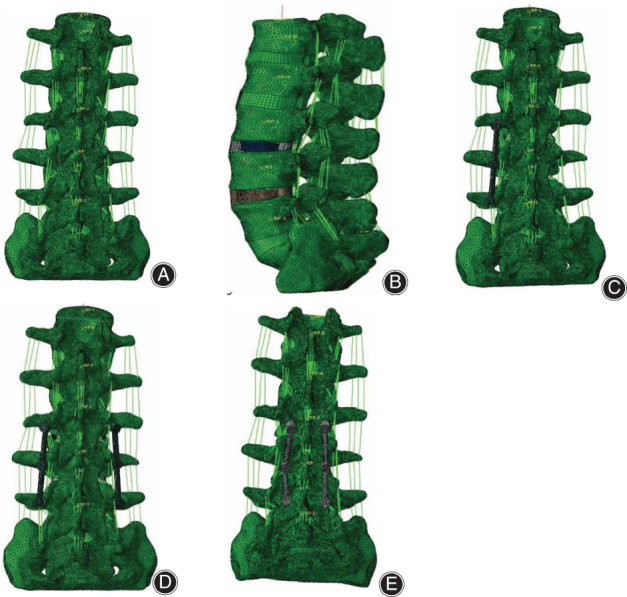
Various finite element models: (A) intact osteoporosis model, (B) two stand‐alone cages, (C) two cages with unilateral pedicle screws, (D) two cages with bilateral pedicle screws, and (E) two cages with bilateral cortical bone trajectory screws

The L3–L5 intervertebral space was used as the surgical segment, and the annulus fibrosus, nucleus pulposus, and cartilage endplate were removed from the left side. Then, we constructed four surgical models: (a) two stand‐alone cages (SA); (b) two cages with UPS; (c) two cages with BPS; and (d) two cages with CBT. The interbody cage was fixed in the same position in all surgical models. Finite element models of various fixations are shown in Figure 1.

### Boundary and Loading Conditions

The inferior surface of the S1 vertebra was fixed so that all nodes of the inferior end plate of the S1 vertebra were constrained from moving in any direction. Then, a vertical load of 400 N^10^ was applied to the upper surface of L1 to simulate the axial load (upright state) of physiological compression, and a torsional moment of 10 Nm was imposed to simulate six different physiological movements of the lumbar spine: flexion, extension, left bending (LB), right bending (RB), left rotation, and right rotation (RR). After numerical calculation, the surgical segment range of motion (ROM) was obtained and compared with the intact osteoporosis model. In addition, the surgical segment ROM, cage stress, and supplemental fixation stress were compared among different internal fixation methods.

### Validation of the Model

**Fig. 2:**
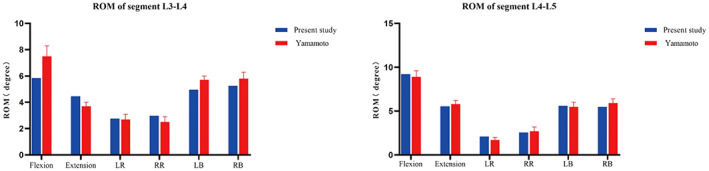
Comparison of the ROM between the normal intact model and the previous in vitro experimental study. LB: left bending; RB: right bending; LR: left rotation; RR: right rotation; ROM: range of motion

The L3–L5 segment ROMs for different motions of the normal intact model were compared with the in vitro experimental data by Yamamoto et al.^24^ As shown in Figure 2, the L3–L5 ROM of the normal intact model was in good agreement with that reported in the literature, which verifies the validity of the intact model.

## Results

### Range of Motion of the Surgical Segment [L3–L5]

**Fig. 3:**
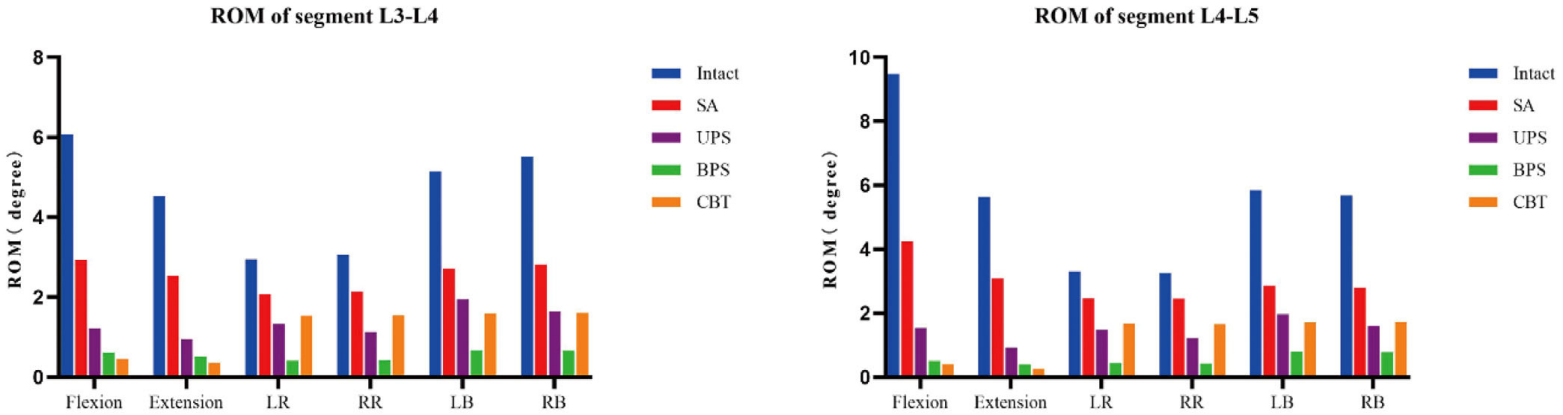
The ROM of segment (L3–L5). Intact: intact osteoporosis model; SA: stand‐alone cage; UPS: cage with unilateral pedicle screws; BPS: cage with bilateral pedicle screws; CBT: cage with bilateral cortical bone trajectory screws; LB: left bending; RB: right bending; LR: left rotation; RR: right rotation; ROM: range of motion

Compared with the ROM of the intact osteoporosis model, the SA model had a minimal reduction in mobility, with reductions of 51.65%–55.16% in flexion, 43.96%–45.20% in extension, 47.50%–50.99% in LB, 48.77%–50.88% in RB, 25.13%–29.57% in LR, and 24.39%–29.95% in RR. In flexion and extension activities, the UPS model decreased by 79.89%–83.71% and 78.91%–83.73%, the BPS model decreased by 89.90%–94.68% and 88.74%–93.01%, and the CBT model decreased by 92.45%– 95.77% and 92.12%–95.40%. The CBT model had the most obvious reduction in flexion and extension activity, and the BPS model was slightly less than the CBT model but larger than the UPS model. For left–right bending and rotation, the UPS model decreased by 62.21%–72.11% and 54.81%–63.20%, the BPS model decreased by 86.27%–87.96% and 86.03%–87.05%, and the CBT model decreased by 69.05%– 70.77% and 47.70%–49.59%, respectively. The BPS model had the greatest limitation in left–right bending and rotation, which was greater than the UPS and CBT models. Compared with the BPS and UPS models, the CBT model had the smallest limitation in left–right rotation. The ROMs of segments L3–L5 in all models are shown in Figure 3.

### Cage Stress [L3–L5]

**Fig. 4:**
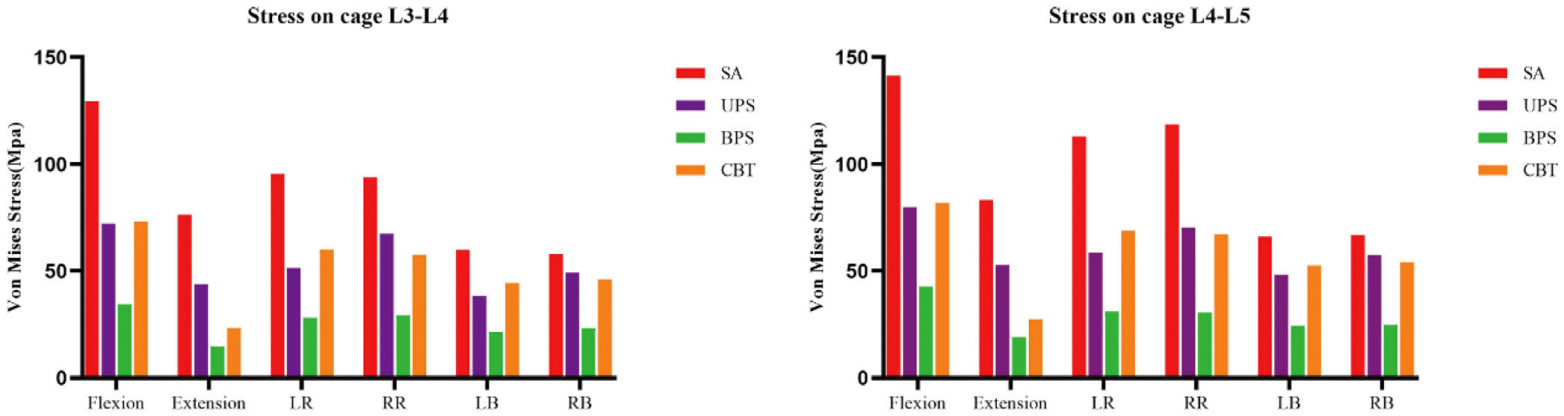
Stress on cage (L3–L5). SA: stand‐alone cage; UPS: cage with unilateral pedicle screws; BPS: cage with bilateral pedicle screws; CBT: cage with bilateral cortical bone trajectory screws; LB: left bending; RB: right bending; LR: left rotation; RR: right rotation

**Fig. 5:**
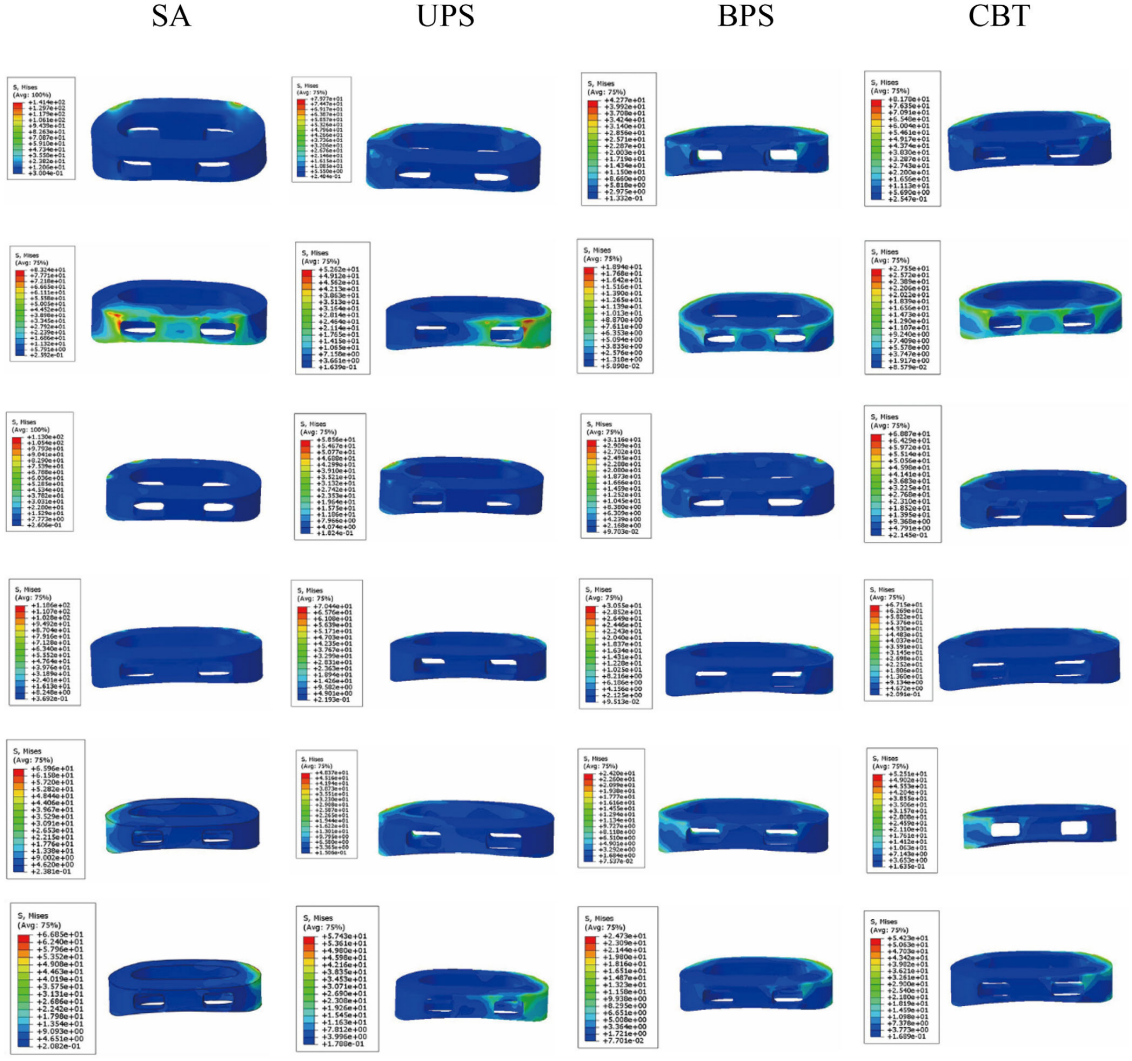
Cage stress distribution was observed in four groups of surgical models during all motions. Pictures of each group of models from top to bottom are flexion, extension, left rotation, right rotation, left bending, and right bending. UPS: cage with unilateral pedicle screws; BPS: cage with bilateral pedicle screws; CBT: cage with bilateral cortical bone trajectory screws

The highest stresses of the SA model during flexion, extension, LB, RB, LR, and RR were 129.30–141.4 MPa, 76.37–83.24 MPa, 59.93–65.96 MPa, 57.93–66.85 MPa, 95.47–113 MPa, and 93.89–118.60 MPa, respectively, which were higher than those of the other surgical models. Among all surgical models, the cage stress in the BPS model was the lowest. Compared with the UPS model, the cage stress in the CBT model was larger in terms of flexion and LB and LR but slightly smaller in terms of RB and RR. In the extension, the cage stress in the CBT model is significantly smaller than in the UPS model. The stress of the cage in the L3–L5 segments is shown in Figure 4. In all motions, stress was distributed outside the cage, as shown in Figure 5.

### Supplemental Fixation Stress[L3‐L5]

**Fig. 6:**
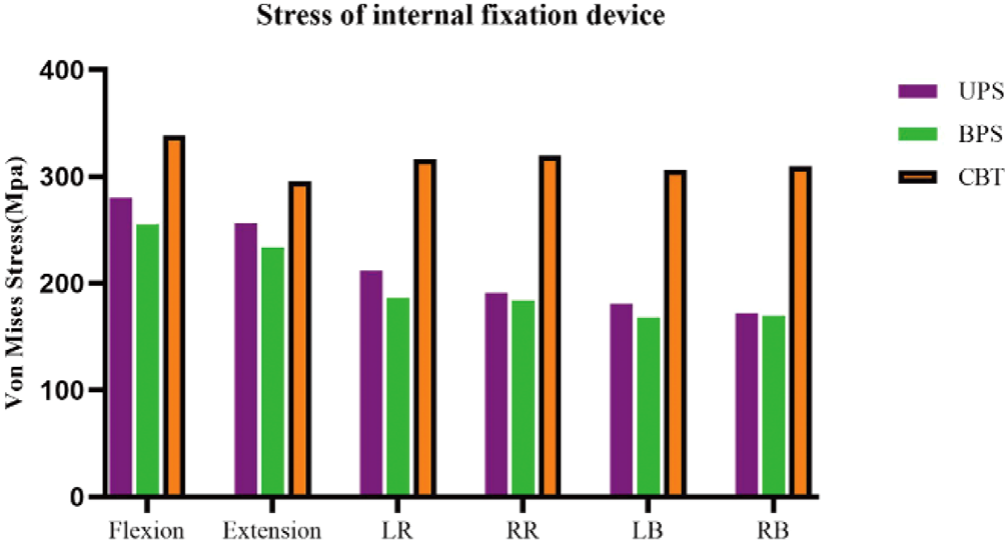
Stress of internal fixation devices. UPS: cage with unilateral pedicle screws; BPS: cage with bilateral pedicle screws; CBT: cage with bilateral cortical bone trajectory screws; LB: left bending; RB: right bending; LR: left rotation; RR: right rotation

**Fig. 7:**
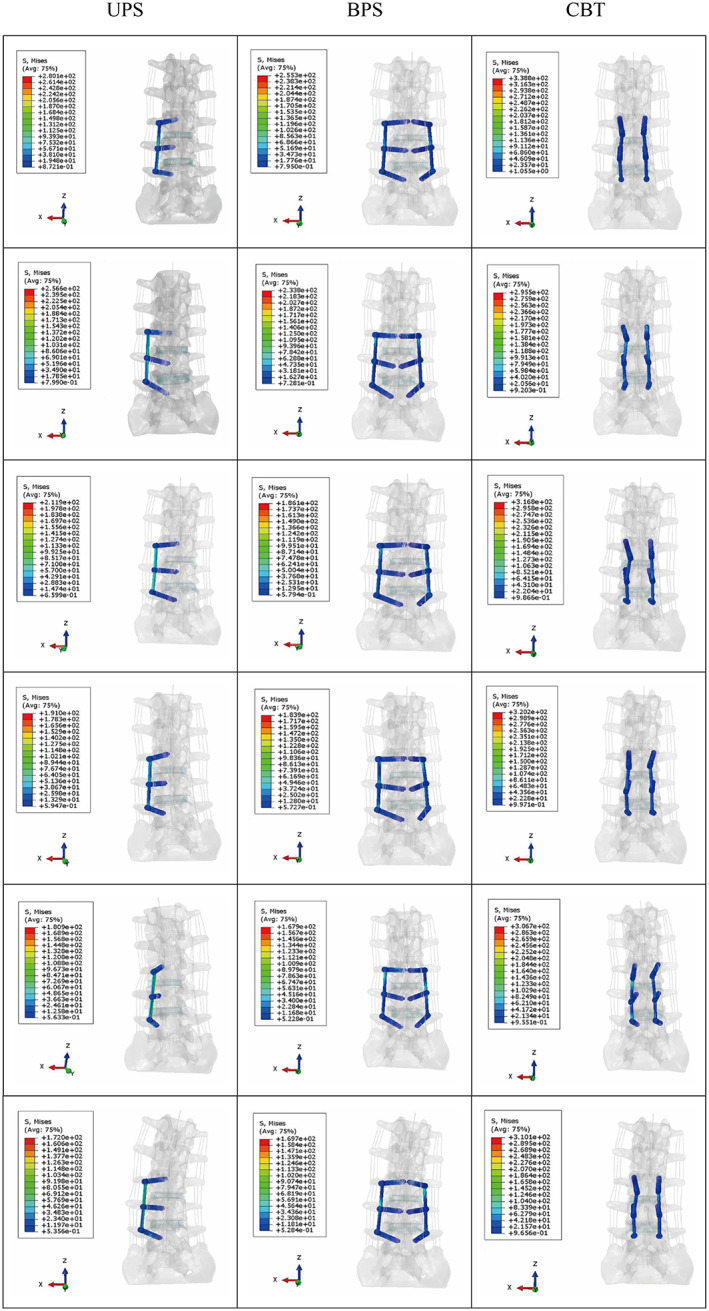
Stress distribution of internal fixation devices. The models of all internal fixation groups from top to bottom are flexion, extension, left rotation, right rotation, left bending, and right bending. UPS: cage with unilateral pedicle screws; BPS: cage with bilateral pedicle screws; CBT: cage with bilateral cortical bone trajectory screws

For the USP model, the internal fixation stress was 280.10 MPa, 256.60 MPa, 180.90 MPa, 172 MPa, 211.90 MPa, and 191 MPa in flexion, extension, LB, RB, LR, and RR, respectively. The internal fixation stress of the BPS model during flexion, extension, LB, RB, LR, and RR was 255.30 MPa, 233.80 MPa, 167.90 MPa, 169.70 MPa, 186.10 MPa, and 183.90 MPa, respectively. For the CBT model, the internal fixation stress was 338.80 MPa, 295.50 MPa, 306.70 MPa, 310.1 MPa, 316.80 MPa, and 320.20 MPa in flexion, extension, LB, RB, LR, and RR, respectively. The CBT internal fixation was subjected to the highest stress of all motions. The BPS group had the lowest internal fixation stress in all motions. The internal fixation stress in all models is shown in Figure 6. The stress of the internal fixation device was concentrated at the junction of the screw and the vertebrae, as shown in Figure 7.

## Discussion

In this study, ROM of segment, cage stress, and internal fixation stress were studied in all surgical models. Our major findings are: (i) The CBT model had the most noticeable reduction in flexion and extension activities, and had the smallest limitation in left–right rotation. The BPS model had the greatest limitation in left–right bending and rotation, which was greater than the UPS and CBT models. Meanwhile, BPS also had obvious restrictions in flexion and extension activities. The BPS model had the lowest internal fixation stress and cage stress in all motions. The CBT internal fixation was subjected to the highest stress of all motions. (ii) The biomechanical performance of BPS model is better than that of CBT model and UPS model.

With the increasing application of OLIF surgery, extensive attention has been given to its complications. As with other traditional fusion procedures, OLIF surgery has a risk of cage subsidence and postoperative segmental instability.^25^, ^26^ Lumbar degenerative diseases are usually associated with two or more degenerative segments in elderly patients with osteoporosis, which often require auxiliary posterior fixation. At present, few studies have used FE analysis methods to research the biomechanical stability of double‐level OLIF with different supplementary fixations.

### Effects of All Surgical Models on ROM of Segment

The purpose of LIF is to increase the segmental stability of the operation. The worse the stability, the higher the incidence of cage subsidence and nonfusion.^27^ Compared to the intact model, our study showed that the SA model had the least decline in ROM across all motion modes, resulting in the least ability to maintain lumbar spine stability. The BPS model had greater limitations than the UPS model in all motions. These results are consistent with the findings of previous studies.^28^, ^29^ The results of this study suggested that, compared with the SA model, all supplemental fixation modalities enhanced the stability of the lumbar spine structure. A literature review by Oxland et al.^30^ found that the addition of posterior instrumentation to the interbody spacer significantly increased structural stability, regardless of cage insertion trajectory or screw type. Matsukawa et al.^31^ conducted a biomechanical finite element study and showed that the CBT method is superior to the BPS in flexion and extension, while the BPS is superior in rotation and bending. Our research also found that the CBT model had the smallest limitation on left–right rotation and the most obvious restriction in flexion and extension. The reason for this result is related to the diameter and length of the screw and the trajectory of screw placement. In clinical studies, the fusion rate of CBTs was lower than that of BPSs, which may be related to fewer restrictions in bending and rotation.^32^ However, the BPS model is only slightly less restrictive to flexion and extension than the CBT model. The stability of the BPS model was stronger than that of the UPS model in all motion states. This is because BPS further increases the stiffness of the operative segment and reduces the coupling motion effect. A cadaveric study of multilevel lateral lumbar interbody fusion (LLIF) found that even in multilevel LLIF surgery, the BPS provided greater stability than UPS.^33^ Therefore, our study suggests that BPS has more advantages in limiting segmental motion.

### Differences in Cage Pressure and Correlation with Cage Subsidence Risk

The increase in pressure on the cage leads to a corresponding increase in end‐plate stress, and the risk of end‐plate collapse and cage subsidence increases. In our study, the cage pressure of the SA model was greater than that of all auxiliary internal fixation models. This indicates that cage subsidence and endplate collapse are at higher risk in stand‐alone surgery. Cheng et al.^34^ reviewed 79 patients who underwent OLIF surgery and found that SA OLFI was significantly associated with cage subsidence. The BPS model had the smallest cage pressure of all surgical models. BPS fixation is a three‐column fixation for spinal stability and has sufficient stiffness so that the cage is subjected to minimal pressure in all directions of motion. A retrospective case study showed that SA OLIF had a greater incidence of cage subsidence than BPS OLIF.^35^ Aoki et al.^36^ conducted a study on 125 patients undergoing transforaminal lumbar interbody fusion and found that the incidence of cage displacement in the UPS group (8.3%) was higher than that in the BPS group (2.1%). The cage pressure of the CBT model was higher than that of the BPS model and UPS model in flexion, which may be caused by the fact that CBT only strengthened the rigidity of the middle and posterior column but failed to fix the anterior column. However, the pressure on cages decreased in extension because the CBT is more rigid than the UPS in bilateral fixation. Interestingly, it was found in our study that cage pressures in the CBT model were larger in LB and LR but slightly smaller in RB and RR compared to the UPS model. This may be due to the inherent imbalance in the operative segment caused by unilateral pedicle fixation, leading to stress redistribution and requiring further investigation.

### Analysis of Stress Difference of Internal Fixation Devices

The stress of internal fixation is related to the loosening and fracture of internal fixation. In the present study, we found that the CBT model had the highest internal fixed stress, while the BPS model had the lowest internal fixed stress. Because the diameter and length of CBT screws are smaller than those of BPS screws, the contact area between the screws and bone is small, which leads to increased pressure. The oblique upwards trajectory of CBT screws is subjected to more concentrated stress. Akpolat et al.^37^ conducted a lumbar cadaver study and found that the standard pedicle screw had better fatigue performance than the CBT screw in vertebrae with compromised bone quality. Matsukawa et al.^38^ concluded in a biomechanical study that increasing the screw diameter and length could reduce the mechanical stress on the bone‐screw interface. Due to the unilateral fixation of UPS, the internal fixation stress is concentrated, resulting in greater internal fixation stress than BPS. Our experimental results showed that regardless of the kind of internal fixation, the pressure was less than the yield strength of titanium alloy 897–1034 MPa.^39^ Based on the results, we suggest that osteoporotic patients are not eligible for stand‐alone OLIF surgery and that patients after CBT‐assisted internal fixation should avoid rotation and bending as much as possible.

### Limitation and Strengths

There are some limitations to this research. First, our model has the common problem of finite element analysis, ignoring the influence of paraspinal muscles on the biomechanical function of the spine, and cannot perfectly replicate the complex bioactive structure of the human body. Second, we did not consider peripheral soft tissue injuries for each surgical mode, which may have an impact on lumbar stability. Third, the results may be influenced by different degrees of osteoporosis, which were not evaluated in this study. Nevertheless, our study has been validated, and the results obtained are in good agreement with previous studies, which have guiding significance for clinical practice. The main strength of this study is its focus on double‐level OLIF. To our knowledge, this may be the first study to evaluate the biomechanical properties of double‐level OLIF with different supplementary fixations. This will provide some reference for future multi‐level OLIF research and even the research of degenerative lumbar scoliosis.

### Conclusions

Supplemental internal fixation can improve segmental stability and lessen cage stress in double‐level OLIF surgery. In limiting segmental mobility and lowering the stress of cage and internal fixation, BPS outperformed UPS and CBT.

#### An Authorship Declaration

All authors listed meet the authorship criteria according to the latest guidelines of the International Committee of Medical Journal Editors. All authors are in agreement with the manuscript.

## Conflict of Interest

The authors declare no conflict of interest.

## Authors' Contributions

Kaibin Fan was responsible for writing the paper, sorting the experimental data, and participating in the experimental design. Di Zhang and Rui Xue participated in data collection and project design. Kaibin Fan, Di Zhang, and Rui Xue contributed equally to this work. Wei Chen, Zhiyong Hou, and Yingze Zhang supported the project and commented on the manuscript. Xianzhong Meng revised the manuscript. All authors agree to the final manuscript.
